# Suppression of Alzheimer's Disease-Like Pathology Progression by Mitochondria-Targeted Antioxidant SkQ1: A Transcriptome Profiling Study

**DOI:** 10.1155/2019/3984906

**Published:** 2019-07-15

**Authors:** Natalia A. Stefanova, Nikita I. Ershov, Nataliya G. Kolosova

**Affiliations:** ^1^Institute of Cytology and Genetics, Novosibirsk, Russia; ^2^Novosibirsk State University, Novosibirsk, Russia

## Abstract

Alzheimer's disease (AD) is the most common type of dementia, with increasing prevalence and no disease-modifying treatment available yet. There is increasing evidence—from interventions targeting mitochondria—that may shed some light on new strategies for the treatment of AD. Previously, using senescence-accelerated OXYS rats that simulate key characteristics of sporadic AD, we have shown that treatment with mitochondria-targeted antioxidant SkQ1 (plastoquinonyl-decyltriphenylphosphonium) from age 12 to 18 months (that is, during active progression of AD-like pathology)—via improvement of mitochondrial function—prevented the neuronal loss and synaptic damage, enhanced neurotrophic supply, and decreased amyloid-*β*_1–42_ protein levels and tau hyperphosphorylation in the hippocampus. In the present study, we continued to explore the mechanisms of the anti-AD effects of SkQ1 in an OXYS rat model through deep RNA sequencing (RNA-seq) and focused upon the cell-specific gene expression alterations in the hippocampus. According to RNA-seq results, OXYS rats had 1,159 differentially expressed genes (DEGs) relative to Wistar rats (control), and 6-month treatment with SkQ1 decreased their number twofold. We found that 10.5% of all DEGs in untreated (control) OXYS rats were associated with mitochondrial function, whereas SkQ1 eliminated differences in the expression of 76% of DEGs (93 from 122 genes). Using transcriptome approaches, we found that the anti-AD effects of SkQ1 are associated with an improvement of the activity of many signaling pathways and intracellular processes. SkQ1 changed the expression of genes in neuronal, glial, and endothelial cells, and these genes are related to mitochondrial function, neurotrophic and synaptic activity, calcium processes, immune and cerebrovascular systems, catabolism, degradation, and apoptosis. Thus, RNA-seq analysis yields a detailed picture of transcriptional changes during the development of AD-like pathology and can point to the molecular and genetic mechanisms of action of the agents (including SkQ1) holding promise for the prevention and treatment of AD.

## 1. Introduction

Alzheimer's disease (AD) is the most common form of dementia in the elderly, with increasing prevalence and no disease-modifying treatment available yet. There is a large amount of data suggesting that oxidative stress plays an important role in the pathogenesis of AD in conjunction with augmentation of protein oxidation, protein nitration, glycol oxidation, and lipid peroxidation as well as accumulation of the amyloid *β* (A*β*) peptide, which can also induce oxidative stress [1]. Nevertheless, no clinical studies conducted to date have proved a beneficial effect of antioxidant treatment in AD patients [2, 3]. For example, vitamin E or selenium used alone or in combination cannot prevent dementia in asymptomatic older men [4]. It has been theorized that inaccessibility of a specific cell compartment to antioxidant agents—where there are increased levels of reactive oxygen species (ROS)—leads to the failure of treatment strategies based on antioxidant supplements. Neuronal homeostasis requires healthy mitochondria. Many lines of evidence suggest that mitochondrial abnormalities as well as the related oxidative stress together with synaptic degeneration are the earliest and most prominent features of vulnerable neurons in the brain of AD patients [5, 6]. AD-related studies have provided substantial evidence on interventions targeting mitochondria, and these data may shed some light on new strategies against AD and suggest that mitochondria-targeted antioxidants hold promise for the treatment of AD [7, 8].

Previously, we have shown that mitochondria-targeted antioxidant plastoquinonyl-decyltriphenylphosphonium (SkQ1) [9] at nanomolar concentrations can prevent, slow down, or partially retard AD-like pathology in accelerated-senescence OXYS rats [10–14], which spontaneously develop all the major signs of AD and largely reproduce the stages of the disease [15–19]. We have shown that dietary supplementation with SkQ1 starting at the preclinical stage of AD-like pathology (at age 1.5 months) reduces the age-related alterations in behavior and the spatial memory deficit and slows down pathological accumulation of A*β* and hyperphosphorylation of tau protein in 23-month-old OXYS rats [11]. Later, we demonstrated that SkQ1 can delay progression of AD signs in OXYS rats [12] starting from the disease stage that we can define as an analog of the predementia phase of AD in humans. Through improvement of the structural and functional state of mitochondria, treatment with SkQ1 from age 12 to 18 months prevented the neuronal loss and synaptic damage, enhanced neurotrophic supply, and decreased A*β*_1–42_ peptide levels and tau hyperphosphorylation in the hippocampus of OXYS rats, thus improving the learning ability and memory. Moreover, recently, we showed that SkQ1 can alleviate some signs of AD-like pathology in OXYS rats even at the stage of severe neurodegenerative damage [14]. It is noteworthy that its prophylactic and therapeutic effects in all cases were associated with improvement of the mitochondrial apparatus. SkQ1 is an antioxidant and has been developed as such to counteract oxidative damage in mitochondria [9]. One would expect that the effects of SkQ1 are mediated by the suppression of reactive oxygen species (ROS) production. Nevertheless, we did not detect enhanced production of ROS by brain mitochondria from 5- and 24-month-old rats: when the AD-like pathology developed and progressed, and well-pronounced structural disturbances in hippocampal mitochondria were observed in OXYS rats [20]. Thus, the specific mechanisms of action of SkQ1 are still unclear. Transcriptomic analyses hold much promise for elucidating the pathogenesis of complex diseases including AD [21] and for genetic assessment of potential therapeutic agents inhibiting the initiation and progression of diseases. In this study, we explored the mechanisms of the anti-AD effects of SkQ1 in a senescence-accelerated OXYS rat model by deep RNA sequencing (RNA-seq) and focused on cell-specific gene expression alterations in the hippocampus.

## 2. Materials and Methods

### 2.1. Compound

SkQ1 was synthesized and provided by the Institute of Mitoengineering of Moscow State University (Moscow, Russia).

### 2.2. Animal Treatments

All the experimental procedures complied with the European Communities Council Directive of 24 November 1986 (86/609/EEC). The protocol of the animal study was approved by the Commission on Bioethics of the Institute of Cytology and Genetics, Novosibirsk, Russia. Male senescence-accelerated OXYS rats and Wistar rats (control) were obtained from the Center for Genetic Resources of Laboratory Animals at the Institute of Cytology and Genetics, the Siberian Branch of the Russian Academy of Sciences (RFMEFI61914X0005 and RFMEFI61914X0010). The OXYS strain has been derived from the rat Wistar strain at the Institute of Cytology and Genetics as described elsewhere [10] and has been registered in the Rat Genome Database (http://rgd.mcw.edu/). Currently, we have the 112th generation of OXYS rats, which spontaneously develop cataract and accelerated-senescence syndrome showing linked inheritance.

At age 4 weeks, the pups were weaned, housed in groups of five per cage (57 × 36 × 20 cm), and kept under standard laboratory conditions (22°C ± 2°C, 60% relative humidity, and 12-hour light/12-hour dark cycle; lights on at 9 a.m.). The animals had *ad libitum* access to standard rodent feed (PK-120-1; Laboratorsnab Ltd., Moscow, Russia) and water.

To assess the influence of oral SkQ1 administration (from age 12 to 18 months) on the progression of AD-like pathology, 12-month-old male OXYS rats were assigned randomly to one of the two groups (*n* = 15). One group consumed a control diet with the addition of dried bread slices, while the other consumed the same diet supplemented with SkQ1 (250 nmol/(kg of body weight)) on the dried bread slices. Each rat in the treatment group received SkQ1 daily. As controls, we employed a group of Wistar rats (*n* = 15). For RNA-seq, three male OXYS rats and three age-matched male Wistar rats at age 18 months were euthanized by CO_2_ asphyxiation. Other animals in each rat group were used for another study [12].

### 2.3. RNA Isolation

After decapitation, the hippocampus was excised rapidly, placed in RNAlater (Ambion, catalog # AM7020), frozen, and stored at −20°C until analysis. Frozen rat tissues were lysed with the TRIzol Reagent (Invitrogen, сat. # 15596–018), and total RNA was isolated. RNA quality and quantity were evaluated on an Agilent Bioanalyzer (Agilent).

### 2.4. Illumina Sequencing

More than 40 million single-end reads 50 bp long were obtained for each sample of hippocampal RNA, by Illumina nonstranded sequencing on an Illumina GAIIx instrument at the Genoanalitika Lab, Moscow (https://www.genoanalytica.ru/), in accordance with standard Illumina protocols (mRNA-Seq Sample Prep Kit, cat. # 1004816). Briefly, polyadenylated mRNA was purified from total RNA via Sera-Mag Magnetic Oligo (dT) beads and then broken into small fragments by means of divalent cations and heating. Using a reverse transcriptase and random primers, we synthesized first- and second-strand cDNAs. The cDNA was processed in an end-repair reaction with T4 DNA polymerase and Klenow DNA polymerase to blunt the termini. An A base was then added to the 3′ end of the blunt phosphorylated DNA fragments, and an Illumina adaptor with a single T overhang at its 3′ end was next ligated to the end of the DNA fragment, for hybridization in a single-read flow cell. After that, a size range of cDNA templates was selected, and these fragments were amplified on a cluster station with Single-Read Cluster Generation Kit v2. Sequencing-by-synthesis at 50-nucleotide length was performed by means of sequencing-by-synthesis v4 reagents on a Genome Analyzer IIx running the SCS2.8 software (Illumina, cat. # FC-940-4001).

### 2.5. Gene Expression Analysis

The sequencing data were preprocessed using the Cutadapt tool (https://cutadapt.readthedocs.io/) to remove adapters and low-quality sequences. The resulting reads were mapped onto the Rnor_5.0 reference genome assembly in the TopHat2 software (https://ccb.jhu.edu/software/tophat/; [22]). The data were then converted into gene count tables by means of ENSEMBL and RefSeq gene annotation data. The resulting tables were subjected to the analysis of differential gene expression in the DESeq2 software (https://bioconductor.org/packages/release/bioc/html/DESeq2.html; [23]). The genes with *p*_adj_ < 0.05 were selected as differentially expressed.

### 2.6. Analysis of the Expression of Genes Related to Mitochondrial Function and Cell-Specific Expression of Genes

The list of genes related to mitochondrial function was compiled by comparing the gene lists in MitoCarta 2.0 (1158 mouse genes, https://www.broadinstitute.org/pubs/MitoCarta [24]), Integrated Mitochondrial Protein Index, IMPI Q3 2017 database (1483 rat genes, http://impi.mrc-mbu.cam.ac.uk/), and rat genome database, RGD (1232 rat genes, https://rgd.mcw.edu/). The list of genes that are selectively expressed in different cell types (neurons, astrocytes, oligodendrocytes, microglia, and endothelial cells) was created on the basis of data on single-cell RNA-seq [25]. The genes with >5 fragments per kilobase of exon model per million reads mapped (FPKM) and fold enrichment >5 in each cell type (and undetectable or underexpressed in other cell types in the hippocampus) were marked as cell specific.

### 2.7. Functional Analysis and Construction of Gene Interaction Networks

To identify the Gene Ontology (GO) terms overrepresented in a differentially expressed gene (DEG) list, the detected DEGs were subjected to functional enrichment analyses by means of the DAVID (http://david.abcc.ncifcrf.gov/summary.jsp) tool. Pathway analysis of the DEGs was conducted with WebGestalt GSAT (http://www.webgestalt.org/option.php) using KEGG pathways (https://www.genome.jp/kegg/). The gene interaction networks were identified on the GeneMANIA web server (http://www.genemania.org/).

## 3. Results and Discussion

### 3.1. SkQ1 Decreased the Number of DEGs Twofold in the Hippocampus of OXYS Rats

**Figure 1:**
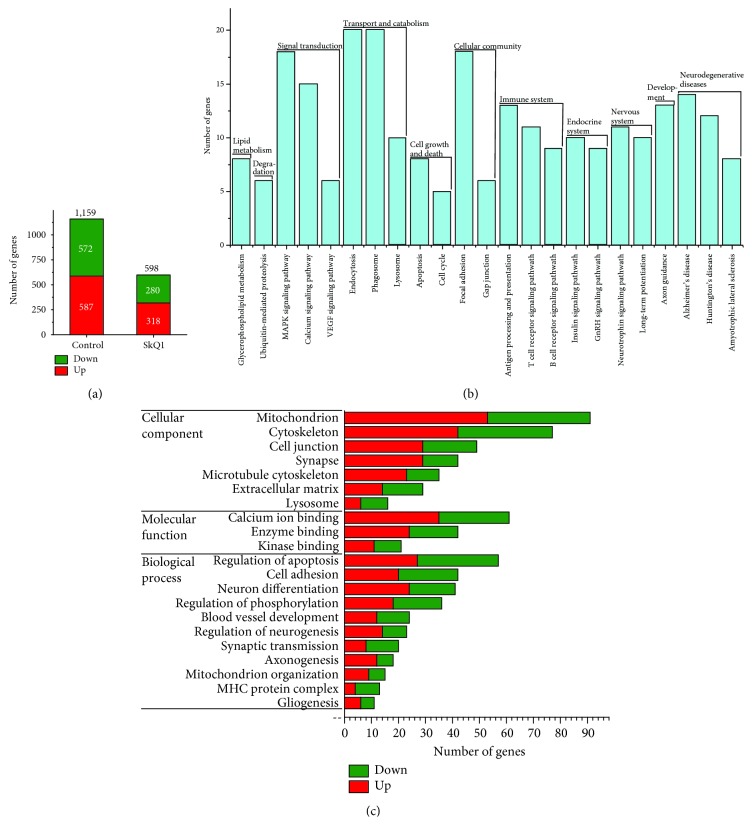
Differential gene expression analysis in the hippocampus of 18-month-old untreated OXYS rats and the influence of long-term treatment with SkQ1. (a) The number of DEGs (*p*_adj_ < 0.05) in control and SkQ1-treated OXYS rats, as compared to Wistar rats, respectively. The number of genes—(b) involved in pathways (according to KEGG) and (c) associated with GO terms (according to DAVID)—that change expression in untreated OXYS rats compared to Wistar rats. GO terms are marked red if upregulated and green if downregulated.

To determine gene expression changes in response to progression of AD-like pathology and for assessment of the anti-AD effects of the mitochondrial antioxidant, we performed RNA-seq on the hippocampal tissue from 18-month-old OXYS and Wistar rats (as a control strain) and OXYS rats treated with SkQ1 from 12 to 18 months of age. A total of 15,911 protein-coding genes with at least 10 counts on average in the hippocampus of OXYS rats and Wistar rats were detected by the DESeq2 software [26]. According to RNA-seq results, 1,159 genes (587 upregulated and 572 downregulated) in the hippocampus of control (untreated) OXYS rats and 598 genes (318 upregulated and 280 downregulated) in the hippocampus of SkQ1-treated OXYS rats were differentially expressed as compared to Wistar rats (*p*_adj_ < 0.05; Figure 1(a)).

### 3.2. Major Altered Biological Processes at the Stage of Well-Pronounced AD-Like Pathology in OXYS Rats

To identify the pathways and processes associated with the DEGs in the hippocampus of control OXYS rats, we carried out gene annotation enrichment analysis using KEGG pathways (*p* < 0.05). We found that at the stage of well-pronounced AD-like pathology in OXYS rats, genes of the calcium signaling pathway, phagosome, endocytosis, axon guidance, gap junction, and apoptosis were up- or downregulated; genes of the GnRH signaling pathway, glycerophospholipid metabolism, and neurodegenerative diseases such as AD, Huntington's disease, and amyotrophic lateral sclerosis were upregulated; genes of the MAPK signaling cascade, insulin signaling, VEGF signal transduction, neurotrophin signaling pathway, long-term potentiation, antigen processing and presentation, T- and B-cell receptor signaling pathways, focal adhesion, ubiquitin-mediated proteolysis, lysosome, and cell cycle were downregulated (Figure 1(b)).

Next, GO analysis of hippocampal DEGs in untreated OXYS rats by DAVID uncovered significant (*p* < 0.05) enrichment of terms related to the cytoskeleton, cell junction, microtubule cytoskeleton, lysosome, Ca^2+^ binding, enzyme binding, kinase binding, cell adhesion, neuron differentiation, and apoptosis, thus indicating significant changes in the transcriptional activity of specific genes participating in neuronal plasticity during AD progression (Figure 1(c)). The enrichment of terms related to the synapse, axonogenesis, and synaptic transmission (Figure 1(c)) pointed to changes in the expression of genes associated with synaptic processes, whose disturbances we revealed recently in the hippocampus of OXYS rats [27]. Aside from terms related to blood vessel development, GO analysis also uncovered significant (*p* < 0.05) enrichment of terms related to major histocompatibility complex (MHC) and gliogenesis (Figure 1(c)), suggesting that in OXYS rats, just as in AD patients [28, 29], the progression of AD-like pathology is associated with changes of the transcriptional activity of genes related to an immune process. Finally, the major altered biological process in OXYS rats was closely related to mitochondrial function; 91 DEGs (53 upregulated and 38 downregulated; Figure 1(c)) were associated with the term *mitochondrion*. Recently, in the hippocampus [20], we found that already at the preclinical stage, OXYS rats manifest some characteristic ultrastructural changes and low activity of respiratory complexes I, IV, and V; these alterations were found to progress with the development of AD-like pathology. In addition, in the prefrontal cortex of OXYS rats, we have previously found that gene expression changes during the development of AD-like pathology (as well as at the preclinical stage) are related to neuronal plasticity, catalytic activity, lipid and immune processes, and mitochondria [18]. Thus, we have previously demonstrated a decrease in mitochondrial function with aging in OXYS rats and an increase in the expression of many mitochondrial genes. Therefore, the results obtained here and previously [13, 15, 18, 20] suggest that mitochondrial dysfunction appears at least to mediate or possibly even initiate pathological molecular cascades of AD-like pathology in OXYS rats.

### 3.3. SkQ1 Restores the Transcriptomic Changes Related to Mitochondrial Function in OXYS Rats

**Figure 2:**
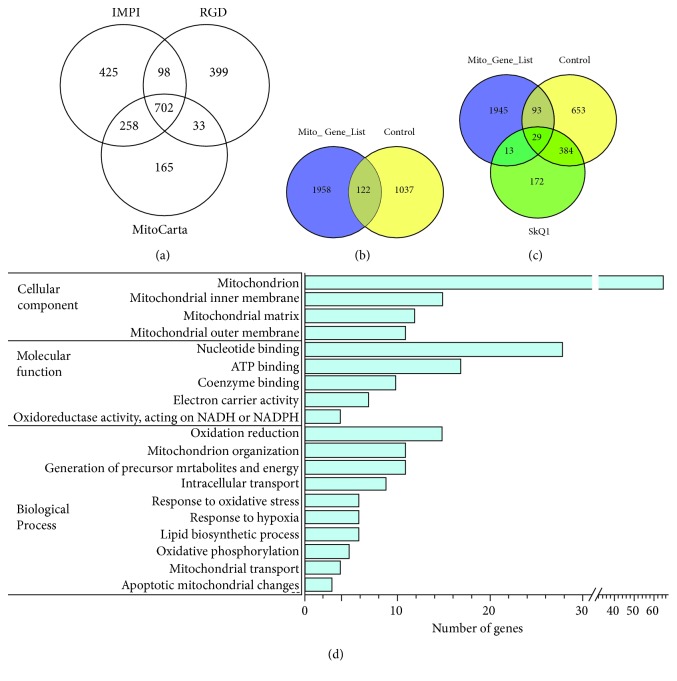
Differential expression of genes related to mitochondrial function in the hippocampus of 18-month-old OXYS rats and the effect of long-term treatment with SkQ1. (a) The Venn diagram shows overlapping sets of genes related to mitochondrial function from MitoCarta 2.0, IMPI Q3 2017, and RGD databases. (b) According to the mitochondrial gene list, the 122 DEGs in OXYS rats are related to mitochondrial function. (c) The Venn diagram suggests that treatment with SkQ1 did not affect the expression of 29 genes in OXYS rats, eliminated differences in the expression of 93 genes, and changed the expression of 13 genes. (d) The number of genes related to mitochondrial function, i.e., associated with GO terms (according to DAVID) that changed expression in control OXYS rats compared to Wistar rats.

Given that 91 DEGs were associated with the term *mitochondrion* according to DAVID, we next performed a more complete analysis of transcriptomic changes associated with the functional category of mitochondria at the stage of well-pronounced AD-like pathology in OXYS rats, using MitoCarta 2.0 (1,158 genes), IMPI (1,483 genes), and RGD (1,232 genes) databases. We compared the genes from these three databases and obtained a combined list of 2,080 genes (Figure 2(a)). We found that 10.5% of DEGs (122 genes) in untreated OXYS rats were associated with mitochondrial function (Figure 2(b) and Supplementary Data 1), confirming the findings that AD-like pathology in OXYS rats is strongly associated with mitochondrial dysfunction. Treatment with SkQ1 eliminated the differences in the expression of 76% of DEGs (93 from 122 genes) and changed the expression of 13 genes in OXYS rats compared to Wistar rats (Figure 2(c) and Supplementary Data 1).

According to DAVID, 122 DEGs related to mitochondrial function in untreated OXYS rats were associated with mitochondrion organization; oxidation-reduction; oxidative phosphorylation; nucleotide, coenzyme, or ATP binding; lipid process; mitochondrial matrix; inner and outer membranes; transport; apoptotic mitochondrial changes; a response to hypoxia and oxidative stress; and others (Figure 2(d)). Oxidative stress and decreased cellular responsiveness to oxidative stress are thought to influence brain aging and AD [30]. Given that changes in the expression of six genes (*Fos*, *Mutyh*, *Jun*, *Bcl2l1*, *Cat*, and *Mmp14*) in the hippocampus of untreated OXYS rats were associated with the GO term “response to oxidative stress,” we performed analyses of genes involved in oxidative stress and antioxidant defense [31]. We identified only three DEGs (*Gsr*, *Cat*, and *Ptgs2*) that participate in these processes at the stage of well-pronounced AD-like pathology in OXYS rats. Metabolic control of oxidation and reduction reactions within the cell is maintained in part by the action of key enzymes including superoxide dismutase, catalase, and glutathione peroxidase and reductase [30]. Among these enzymes, glutathione reductase (*Gsr*) turned out to be upregulated (log_2_fold change = 0.37; *p* < 0.03), and the expression of catalase (*Cat*), which is also crucial for the modulation of synaptic plasticity in the brain [32], was lower (log_2_fold change = −0.52; *p* < 0.015) in OXYS rats. The expression of prostaglandin-endoperoxide synthase 2 (*Ptgs2*), also known as cyclooxygenase 2 (COX-2), which is considered a candidate gene of AD [33, 34], was lower in OXYS rats (log_2_fold change = −0.68; *p* < 0.0005).

These results are consistent with the reports that the development of AD signs in OXYS rats happens before the increased accumulation of oxidative stress markers in the brain [35, 36]. It should be noted that most of the *in vivo* data—suggesting significant contribution of oxidative stress in the brain to the AD pathophysiology—have been obtained in animal models, first of all in transgenic mouse models of the familial form of AD. Our results are consistent with the findings of a recent meta-analysis which was conducted to define the pattern of changes in oxidative stress-related markers in human AD and mild cognitive impairment, by the brain region [30]. Its authors concluded that the antioxidant enzyme system in the brain is largely intact in AD and the global accumulation of oxidative damage is less substantial than generally thought. The limitation of our study is that gene expression changes were not confirmed at the protein level.

To determine the effects of SkQ1 as a mitochondria-targeted antioxidant on the gene expression profile related to mitochondrial function, we compared the GO terms and genes associated with them between control and SkQ1-treated OXYS rats. We found that prolonged treatment with SkQ1 prevented the changes in the processes related to mitochondrion organization, generation of precursor metabolites and energy, oxidation-reduction, oxidative phosphorylation, mitochondrial transport, apoptosis, and others (Table 1). Recently, we demonstrated that SkQ1 is localized to cerebral neuronal mitochondria and retards mitochondrial alterations in the hippocampus of 18-month-old OXYS rats [12], which we also subjected to the present RNA-seq analysis (see Materials and Methods). SkQ1 treatment substantially improved the ultrastructure of the mitochondrial apparatus, increased the specific area of mitochondria in neurons, and increased the expression of DRP1 (dynamin-1-like protein; mitochondria in fission) and MFN2 (mitofusin 2; mitochondria in fusion) and enzymatic activity of complexes I and IV in the hippocampus of OXYS rats to the level of Wistar rats (control) [12]. In addition, here, 13 genes—whose expression SkQ1 changed in OXYS rats as compared to Wistar rats—turned out to be related to mitochondria, including the mitochondrial inner membrane.

**Figure 3:**
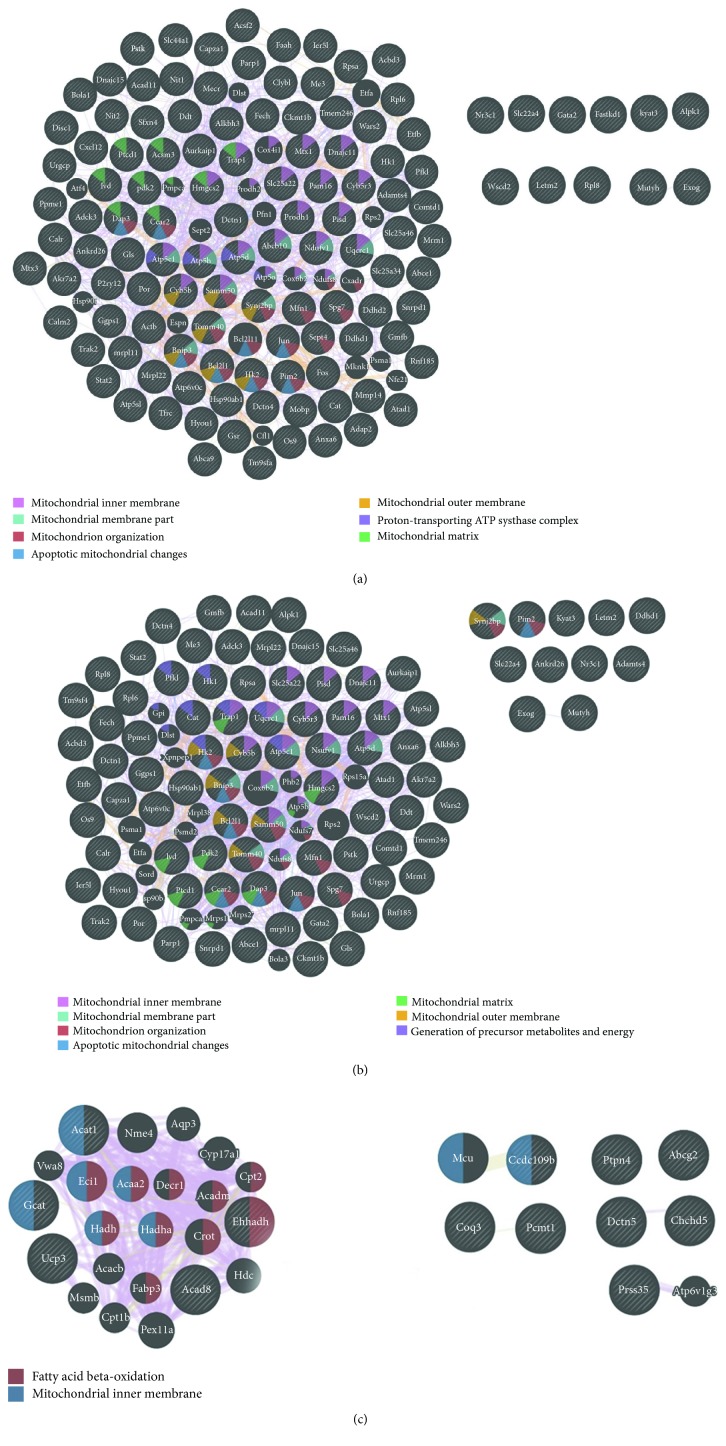
Interaction networks for DEGs related to mitochondrial function in the hippocampus of 18-month-old OXYS rats and the effect of long-term treatment with SkQ1. (a) An interaction network for DEGs in untreated OXYS rats. Interaction networks for DEGs (b) eliminated and (c) changed by SkQ1.

We next used GeneMANIA to build interaction networks for the analysis of transcriptomic changes associated with functional categories of mitochondrial dysfunction at the stage of well-pronounced AD-like pathology in OXYS rats and effects of treatment with SkQ1. In control OXYS rats, some of the most enriched GO terms were the mitochondrial inner membrane (*p* < 1.82*E*‐13), mitochondrial membrane part (*p* < 2.39*E*‐8), mitochondrion organization (*p* < 8.34*E*‐8), apoptotic mitochondrial changes (*p* < 7.87*E*‐4), mitochondrial matrix (*p* < 9.96*E*‐4), mitochondrial outer membrane (*p* < 5.25*E*‐3), and the proton-transporting ATP synthase complex (*p* < 9.57*E*‐3; Figure 3(a)). SkQ1 affected all these processes (Figure 3(b)) and changed the expression of genes related to fatty acid *β* oxidation (*p* < 4.43*E*‐15) and mitochondrial inner membrane (*p* < 1.15*E*‐5; Figure 3(c)).

### 3.4. SkQ1 Retarded the Alteration of Major Biological Processes and Signaling Pathways during the Progression of AD-Like Pathology in OXYS Rats

Recently, using RNA-seq analysis, we demonstrated that mitochondrial impairments in the prefrontal cortex of OXYS rats are the earliest change and progress with age along with the age-related lipid and immune aberrations, cerebrovascular alterations, and increased neuronal degeneration [18]. Thus, we decided to determine the potential mechanisms of action of SkQ1 that may be linked to its effects (activation or suppression of signaling pathways and processes, aside from improvements in mitochondrial processes). To this end, we next performed functional annotation clustering via DAVID and pathway analysis by means of KEGG on DEGs exclusively expressed in the hippocampus of control and SkQ1-treated OXYS rats.

**Figure 4:**
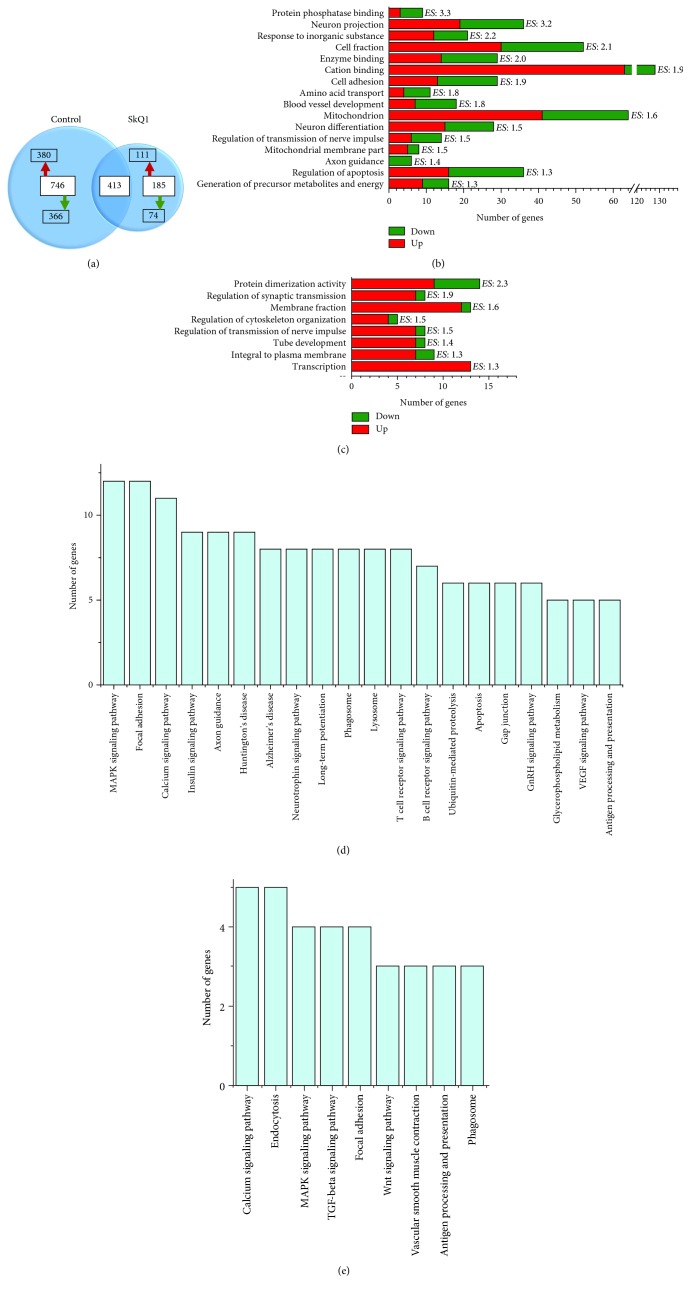
Gene distribution and enrichment analysis of 18-month-old untreated OXYS rats and OXYS rats treated with SkQ1. (a) An area-proportional Venn diagram, summarizing gene identifiers in the overlap (413 genes) between untreated and SkQ1-treated OXYS rats. The analysis of gene distribution points to specific changes in the expression of 746 genes in untreated OXYS rats, 380 of which are upregulated (red arrow) and 366 downregulated (green arrow). OXYS rats treated with SkQ1 showed specific changes in the expression of 185 genes, 111 of which were upregulated (red arrow) and 74 downregulated (green arrow). DAVID cluster analysis performed on DEGs exclusively expressed in the hippocampus of (b) untreated and (c) SkQ1-treated OXYS rats identified the most statistically significant upregulated and downregulated biological processes. The significance of each gene cluster is evaluated through the enrichment score (ES). An ES >1.3 is equivalent to a nonlog scale value of 0.05. The pathway analysis by KEGG for DEGs was exclusively expressed in the hippocampus of (d) untreated and (e) SkQ1-treated OXYS rats.

The two sets of DEGs (untreated OXYS rats versus Wistar rats; SkQ1-treated OXYS rats versus Wistar rats) overlapped, and the overlap contained 413 genes (Figure 4(a)), suggesting that SkQ1 did not affect their expression. Treatment with SkQ1 effected all major altered biological processes during the progression of AD-like pathology; only in untreated OXYS rats, 746 genes exclusively showing upregulation (380 genes) and downregulation (366 genes; Figure 4(a)) were mainly related to mitochondrial function, cation binding, enzyme binding, neuron projection and differentiation, cell adhesion, blood vessel development, regulation of apoptosis, and other processes, according to DAVID (Figure 4(b)). Moreover, SkQ1 changed the expression of 185 genes (111 upregulated and 74 downregulated) in the hippocampus of OXYS rats as compared to untreated OXYS and Wistar rats (Figure 4(a)). These exclusively expressed genes were mainly related to the upregulation of the processes associated with transcription, synaptic processes, tube development, and a membrane part (Figure 4(c)).

Moreover, treatment with SkQ1 affected all key signaling pathways altered during the progression of AD-like pathology. According to KEGG, DEGs exclusively expressed in the hippocampus of untreated OXYS rats were associated with MAPK, calcium, insulin, GnRH, VEGF, and neurotrophin signaling pathways; focal adhesion; synaptic processes (long-term potentiation, axon guidance); immune system (T- and B-cell receptor signaling pathways, antigen processing, and presentation); catabolism (phagosome, lysosome); degradation (ubiquitin-mediated proteolysis); apoptosis; and neurodegenerative diseases (Huntington's disease and AD; Figure 4(d)). In addition, in SkQ1-treated OXYS rats, exclusively expressed genes were mainly related to MAPK, calcium, TGF-*β*, and Wnt signaling pathways; endocytosis; focal adhesion; phagosome; vascular smooth muscle contraction; and antigen processing and presentation (Figure 4(d)).

### 3.5. Effects of SkQ1 on the Cell-Specific Gene Expression in the Hippocampus of OXYS Rats

**Figure 5:**
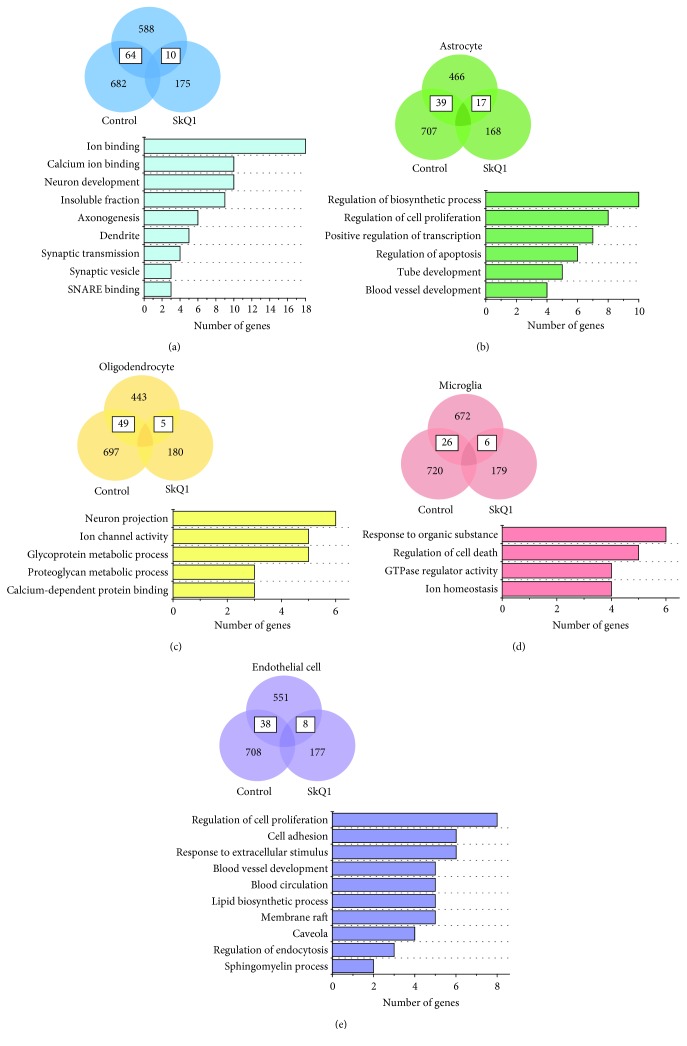
Cell-specific gene distribution and enrichment analysis in the hippocampus of 18-month-old untreated and SkQ1-treated OXYS rats. Venn diagrams and enrichment analyses by DAVID for (a) neurons, (b) astrocytes, (c) oligodendrocytes, (d) microglia, and (e) endothelial cells in untreated and SkQ1-treated OXYS rats.

For a more complete understanding of which cell type(s) and cellular processes represent the most significant effects of SkQ1 during the progression of AD-like pathology, we next performed analyses of the cell-specific gene expression [25] on DEGs exclusively expressed in the hippocampus of untreated and SkQ1-treated OXYS rats. We found that in OXYS rats, treatment with SkQ1 eliminated the differences in the expression of 64 genes (49 upregulated and 15 downregulated) specific to neurons, 39 genes (11 upregulated and 28 downregulated) specific to astrocytes, 49 genes (18 upregulated and 31 downregulated) specific to oligodendrocytes, 26 genes (19 upregulated and seven downregulated) specific to microglia, and 38 genes (eight upregulated and 30 downregulated) specific to endothelial cells (Figures 5(a)–5(e) and Supplementary Data 1). In addition, treatment with SkQ1 changed the expression of 10 genes (upregulated seven and downregulated three) specific to neurons, 17 genes (15 upregulated and two downregulated) specific to astrocytes, five genes (two upregulated and three downregulated) specific to oligodendrocytes, six genes (five upregulated and one downregulated) specific to microglia, and eight genes (five upregulated and three downregulated) specific to endothelial cells relative to Wistar rats (Figures 5(a)–5(e) and Supplementary Data).

For the functional analyses, we combined the lists of eliminated and exclusively changed up- and downregulated DEGs in OXYS rats. DAVID revealed that in neurons (74 genes), the major effects of SkQ1 were associated with ion binding (e.g., *Scn2b*, *Zic1*, *Atp2b2*, *Nptxr*, *Eno2*, *Gucy1a2*, and *Kcnq2*), including calcium ion binding (e.g., *Calb1* and *Calb2*), neuron development (e.g., *Gprin1*, *Atp2b2*, *Epha7*, and *Unc5a*), and synaptic GO terms such as axonogenesis (*Epha7*, *Ndn*, *Unc5a*, *Stxbp1*, *Rtn4r*, and *Snap25*), dendrite (*Atp2b2*, *Epha7*, *Inpp5j*, *Crmp1*, and *Cpne6*), regulation of synaptic transmission (*Syp*, *Atp2b2*, *Stxbp1*, and *Calb1*), synaptic vesicle (*Syp*, *Rab3b*, and *Svop*), and SNARE (soluble NSF attachment receptor) binding (*Syp*, *Stxbp1*, and *Snap25*; Figure 5(a)). Indeed, the progression of AD-like pathology in OXYS rats is linked with substantial structural and functional alterations of neurons and synapses and with their decreased density [27, 37]. Many studies have confirmed that mitochondrial dysfunction is likely to be the leading cause of synaptic loss and neuronal death by apoptosis, especially in brain regions involved in learning and memory, such as the hippocampus [5]. On the basis of the results obtained here and as we reported recently [12], we can draw the conclusion that treatment with SkQ1 possibly facilitated activation of the remaining undamaged neurons and synapses and improved intraneuronal processes, including mitochondrial function. It has been proposed that synaptic mitochondria are more sensitive to damage than nonsynaptic mitochondria, indicating an earlier decrease in mitochondrial trafficking and respiratory function [6]. This notion is supported by SkQ1-induced changes in the expression of hippocampal genes in OXYS rats in relation to mitochondrial and synaptic processes as well as an increased number of excitatory synapses and active zones of synaptic contact and upregulation of pre- and postsynaptic proteins synapsin I and PSD-95 [12], whose downregulation is considered an indicator event in AD [36]. In addition, the normalization of the balance of the neurotrophic supply in the hippocampus of SkQ1-treated OXYS rats [12] may reflect activation of cellular processes promoting the growth of neurites, formation of new synapses, and neuronal survival. In favor of this notion is also a SkQ1-driven decrease in the *Bcl11A-L* expression (B-cell lymphoma 11A-long), a zinc finger transcription factor that is an important downstream effector of glutamate receptors for the regulation of axonal and dendritic arborization. Overexpression of Bcl11A-L counteracts the influence of glutamate receptors on axonal branching and dendritic outgrowth [38]. Another beneficial action of SkQ1 on neuronal activity may be related to the influence on calcium processes, dysregulation of which is important for destabilization of synaptic processes in aging and in the AD brain [39]. Treatment with SkQ1 downregulated genes *Calb1* (calbindin 1, 28 kDa) and *Calb2* (calbindin 2, 29 kDa, calretinin), whose products act as calcium buffers and sensors. In addition, SkQ1 downregulated the *Hpca* (hippocalcin) gene, which is also regarded as a calcium sensor, may participate in general neuronal endocytosis, and performs a critical calcium-sensing function in NMDA receptor- (NMDAR-) mediated hippocampal long-term depression [40].

Accumulated evidence suggests that glial cells, including astrocytes and microglia, mediating immune responses in the brain, are critically important for the maintenance of normal synaptic functioning [41, 42] and play a crucial part in the AD pathogenesis [28, 29]. For astrocytes (56 genes), the major effects of SkQ1 were associated with the regulation of various processes such as a biosynthetic process (e.g., *Plagl1*, *Hes1*, *Jun*, *Nr4a1*, *Nr4a3*, *Abca1*, and *Sox9*), cell proliferation (e.g., *Egfr*, *Hes1*, *Arhgap5*, and *Sox9*), transcription (*Plagl1*, *Hes1*, *Myo6*, *Jun*, *Nr4a1*, *Nr4a3*, and *Sox9*), apoptosis (*Egfr*, *Steap3*, *Jun*, *Nr4a1*, *Bdkrb2*, and *Sox9*), tube development (e.g., *Hes1*, *Rgma*, and *Nr4a3*), and blood vessel development (*Hey2*, *Ppap2b*, and *Cyr61*; Figure 5(b)). For the oligodendrocytes (54 genes), the major actions of SkQ1 were associated with neuron projection (*S100a4*, *Kcnd3*, *Ermn*, *Chrna4*, *Strn*, and *Gria3*), ion channel activity (*Kcnd3*, *Kcna1*, *Chrna4*, *Gria3*, and *Kcnk1*), glycoprotein metabolic process (*Trak2*, *Chst8*, *Acan*, *Dcn*, and *Adamts4*), including a proteoglycan metabolic process (*Chst8*, *Acan*, and *Dcn*), and calcium-dependent protein binding (*S100a4*, *Masp1*, and *Strn*; Figure 5(c)).

For the microglia (32 genes), the effects of SkQ1 were found to be related to a response to an organic substance (*Egr1*, *Cebpa*, *Slc11a1*, *Egr2*, *Dusp1*, and *Bcl2l1*), regulation of cell death (*Pik3cg*, *Cebpb*, *Dusp1*, *Ubc*, and *Bcl2l1*), GTPase regulator activity (*Arhgap4*, *Tbc1d10a*, *Rgs14*, and *Arhgdib*), and ion homeostasis (*Slc11a1*, *Egr2*, *Nab2*, and *Bcl2l1*; Figure 5(d)). Furthermore, among the most highly enriched genes (FPKM >20) for microglia, SkQ1 affected the expression of *Egr1* and *Egr2* (early growth response 1 and 2; Supplementary Data 1), which are transcription factors that belong to the Egr family, participate in an immune response [43], and positively correlate with disease progression in AD [44]. Recently, it was reported that a proinflammatory phenotype of A*β* plaque-associated microglia isolated from 5XFAD mice is related to the upregulation of *Egr2* [45]. Treatment with SkQ1 downregulated the *Egr2* gene and upregulated the *Egr1* gene, whose expression decreases with aging in the rat hippocampus [46]. In addition, SkQ1 decreased the expression of *Nab2* (Ngfi-A-binding protein 2), which in part modulates the activity of EGR1 and EGR2 and is considered a mediator of A*β*-induced neurotoxicity and tau hyperphosphorylation [47]. Thus, it can be assumed that the SkQ1-driven decrease in the A*β*_1–42_ level and tau hyperphosphorylation in the hippocampus of OXYS rats [12] can be related to changes in the expression of microglial genes *Egr1*, *Egr2*, and *Nab2* and activation of phagocytosis by microglia, whose significant ultrastructural abnormalities we uncovered recently [27]. The improvement in phagocytic activity is also supported by an SkQ1-induced increase *Pik3cg* expression (phosphatidylinositol-4,5-bisphosphate 3-kinase catalytic subunit gamma, also known as PI3K*γ*, a specific PI3K isoform; Supplementary Data 1). PI3K*γ*-deficient microglia are strongly associated with decreased phagocytic activity [48] and have a crucial and specific function in NMDAR-mediated synaptic plasticity and some forms of cognitive function [49].

Cerebrovascular dysfunction is strongly involved in the pathogenesis of AD; patients with this disease frequently show focal changes in brain microcirculation. These changes include alterations in the density and morphology of cerebral microvasculature, increased endothelial pinocytosis, decreased mitochondrial content, accumulation of collagen and perlecans in the basement membrane, loss of tight junctions and/or adherens junctions, and a blood–brain barrier breakdown with leakage of blood-borne molecules [50, 51]. According to a magnetic resonance imaging study, 12-month-old OXYS rats, when AD is progressing, undergo structural and functional alterations in cerebral blood flow typical of chronic ischemia [52, 53]. We found that OXYS rats possess altered hemorheological properties of blood resulting from abnormal red blood cell deformability and aggregation [54], which are regarded as some of the mechanisms underlying the complex etiology of AD [55], thereby impairing the oxygen transport efficiency of blood in AD. Recently, we demonstrated that changes in the expression of the hippocampal genes functionally associated with cerebrovascular processes already in the early period of life may contribute to the development of AD-like pathology in OXYS rats [17]. At the advanced stage of the disease in OXYS rats, we observed a significant loss of hippocampal blood vessel density as well as ultrastructural changes and downregulation of VEGF with an increased amount of A*β*_1–42_ in blood vessels. In addition, DEGs in the hippocampus of OXYS rats were associated with downregulation of cerebrovascular function as compared to Wistar rats [17]. Here, for the endothelial cells (46 genes), the effects of SkQ1 turned out to be related to the regulation of cell proliferation (*Cav2*, *Edn3*, *Cav1*, *Ptgs2*, *Crip2*, *Ptges*, *Tek*, and *Cdh5*), cell adhesion (*Ptprk*, *Igfbp7*, *Tek*, *Itga1*, *Cd2ap*, and *Cdh5*), a response to an extracellular stimulus (*Cav1*, *Hmgcs2*, *Ptgs2*, *Ptges*, *Igfbp7*, and *Tek*), a lipid biosynthetic process (*Hmgcs2*, *Ptgs2*, *Sgms2*, *Ptges*, and *Sgms1*), caveola (*Cav2*, *Cav1*, *Ptgs2*, and *Ptrf*), regulation of endocytosis (*Gata2*, *Cav1*, and *Cd2ap*), sphingomyelin biosynthetic process (*Sgms2* and *Sgms1*c), blood vessel development (*Cav1*, *Lama4*, *Dll4*, *Tek*, and *Cdh5*), and circulation (*Edn3*, *Cav1*, *Fli1*, *Ptgs2*, and *Itga1*; Figure 5(e)). Thus, it can be assumed that SkQ1 had a positive effect on the microvascular environment and consequently on the brain's supply of oxygen, energy, substrates, and nutrients.

**Figure 6:**
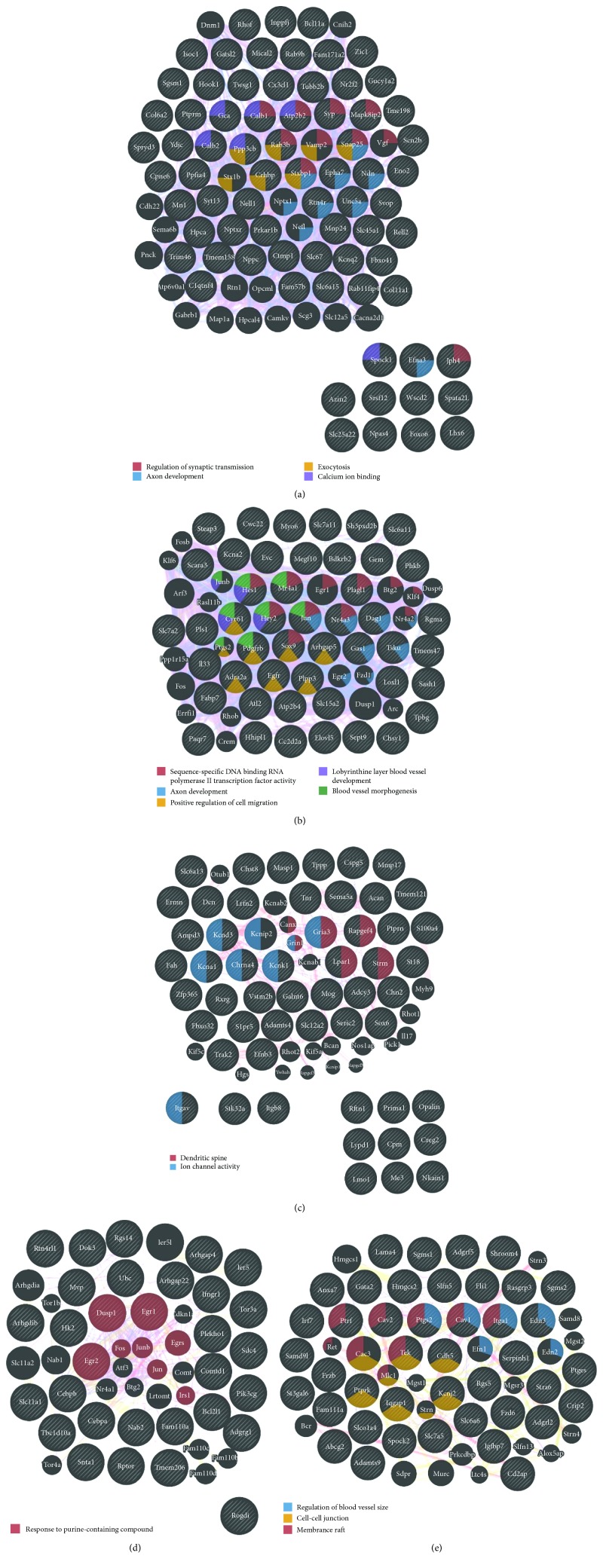
Interaction networks for cell-specific DEGs affected by SkQ1 in the hippocampus of 18-month-old OXYS rats. Interaction networks according to GeneMANIA for DEGs eliminated and changed by SkQ1 in (a) neurons, (b) astrocytes, (c) oligodendrocytes, (d) microglia, and (e) endothelial cells.

Finally, by means of GeneMANIA, we constructed interaction networks for the eliminated and exclusively changed DEGs for the five types of cells in the hippocampus of OXYS rats (Figure 6). We found that GO terms enriched in neurons were related to the regulation of synaptic transmission (*p* < 2.49*E*‐3) and axon development (*p* < 3.85*E*‐2; Figure 6(a)). In astrocytes, the most enriched GO terms were associated with transcription activity (*p* < 2.45*E*‐5), axon development (*p* < 7.42*E*‐3), regulation of cell migration (*p* < 1.15*E*‐3), blood vessel development (*p* < 9.82*E*‐4), and morphogenesis (*p* < 5.42*E*‐3; Figure 6(b)). Among the GO terms enriched in oligodendrocytes, there were dendritic spine (*p* < 2.63*E*‐2) and ion channel activity (*p* < 3.57*E*‐2; Figure 6(c)); in microglia, there was a response to a purine-containing compound (*p* < 8.89*E*‐5; Figure 6(d)); in endothelial cells, there was regulation of blood vessel size (*p* < 2.09*E*‐3), cell-cell junction (*p* < 1.41*E*‐3), and membrane raft (*p* < 1.19*E*‐4; Figure 6(e)).

## 4. Conclusion

In this study, we showed that long-term treatment with SkQ1, an antioxidant specifically targeting mitochondria, starting from the predementia phase of AD signs (consistent with the definition of progressive, amnestic mild cognitive impairment in humans [56]) suppressed the progression of AD-like pathology in OXYS rats via improvement (in neuronal, glial, and endothelial cells) of the gene expression related to mitochondrial function, neurotrophic and synaptic activities, calcium processes, immune and cerebrovascular systems, catabolism, degradation, and apoptosis. Here, we confirmed the previously reported finding that the progression of AD-like pathology in OXYS rats is associated with mitochondrial dysfunction but not associated with oxidative stress. Using transcriptome approaches, we revealed that the anti-AD effects of SkQ1 are not directly related to its antioxidant activity but are associated with an improvement in the functioning of many signaling pathways and intracellular processes. In summary, our results indicate that RNA-seq analysis yields a detailed picture of transcriptional changes during the development of pathological events and may allow researchers to elucidate the molecular and genetic mechanisms of action of promising agents (including SkQ1) for the prevention and treatment of AD.

## Figures and Tables

**Table 1:** The mitochondrial function-related genes (i.e., associated with relevant GO terms according to DAVID) that change the expression in untreated (control) OXYS rats; the effect of prolonged treatment with SkQ1. Differential expression means a comparison with the control Wistar strain.

	Process	Number of DEGs in control OXYS rats	Percentage of DEGs removed by SkQ1	Gene symbols
BP	Mitochondrion organization	11	91	*Sept4*, *Mfn1* ^∗^, *Spg7* ^∗^, *Samm50* ^∗^, *Jun* ^∗^, *Mtx1* ^∗^, *Tomm40* ^∗^, *Bnip3* ^∗^, *Bcl2l1* ^∗^, *Synj2bp* ^∗^, *Dap3* ^∗^
	Generation of precursor metabolites and energy	11	91	*Atp6v0c* ^∗^, *Atp5d* ^∗^, *Uqcrc1* ^∗^, *Pfkl* ^∗^, *Hk2* ^∗^, *Atp5c1* ^∗^, *Hk1* ^∗^, *Cyb5b* ^∗^, *Cat* ^∗^, *Atp5h*, *Etfb* ^∗^
	Oxidation reduction	15	80	*Cyb5r3* ^∗^, *Me3* ^∗^, *Uqcrc1* ^∗^, *Cyb5b* ^∗^, *Por* ^∗^, *Gsr*, *Ivd* ^∗^, *Ndufv1* ^∗^, *Akr7a2* ^∗^, *Cat* ^∗^, *Alkbh3* ^∗^, *Acad11* ^∗^, *Mecr*, *Etfb* ^∗^, *Prodh*
	Oxidative phosphorylation	5	80	*Atp6v0c* ^∗^, *Atp5d* ^∗^, *Uqcrc1* ^∗^, *Atp5c1* ^∗^, *Atp5h*
	Mitochondrial transport	4	100	*Mtx1* ^∗^, *Tomm40* ^∗^, *Bnip3* ^∗^, *Bcl2l1* ^∗^
	Apoptotic mitochondrial changes	3	100	*Jun* ^∗^, *Bcl2l1* ^∗^, *Dap3* ^∗^

MF	Coenzyme binding	10	90	*Cyb5r3* ^∗^, *Gsr*, *Acbd3* ^∗^, *Me3* ^∗^, *Ivd* ^∗^, *Ndufv1* ^∗^, *Cat* ^∗^, *Parp1* ^∗^, *Acad11* ^∗^, *Por* ^∗^
	Nucleotide binding	28	71	*Hsp90ab1* ^∗^, *Cyb5r3* ^∗^, *Sept4*, *Me3* ^∗^, *Fastkd1*, *Spg7* ^∗^, *Abca9*, *Hk2* ^∗^, *Hk1* ^∗^, *Acsf2*, *Gsr*, *Ivd* ^∗^, *Slc22a4* ^∗^, *Abcb10*, *Cat* ^∗^, *Actb*, *Pdk2* ^∗^, *Alpk1* ^∗^, *Pfkl* ^∗^, *Por* ^∗^, *Atad1* ^∗^, *Acsm3*, *Trap1* ^∗^, *Hyou1* ^∗^, *Mfn1* ^∗^, *Ndufv1* ^∗^, *Acad11* ^∗^, *Parp1* ^∗^
	Electron carrier activity	7	86	*Cyb5r3* ^∗^, *Gsr*, *Ivd* ^∗^, *Cyb5b* ^∗^, *Acad11* ^∗^, *Etfb* ^∗^, *Por* ^∗^

CC	Mitochondrion	66	76	*Hsp90ab1* ^∗^, *Cyb5r3* ^∗^, *Atp5d* ^∗^, *Spg7* ^∗^, *Uqcrc1* ^∗^, *Aurkaip1* ^∗^, *Nit1*, *Nit2*, *Bnip3* ^∗^, *Clybl*, *Acsf2*, *Gsr*, *Acbd3* ^∗^, *Dnajc15* ^∗^, *Mutyh* ^∗^, *Dnajc11* ^∗^, *Slc25a22* ^∗^, *Akr7a2* ^∗^, *Exog* ^∗^, *Abcb10*, *Cat* ^∗^, *Atp5h*, *Abce1* ^∗^, *Fech* ^∗^, *Pisd* ^∗^, *Synj2bp* ^∗^, *Cyb5b* ^∗^, *Mrm1* ^∗^, *Por* ^∗^, *Trap1* ^∗^, *Mfn1* ^∗^, *Slc25a34*, *Atp5c1* ^∗^, *Mecr*, *Prodh*, *Me3* ^∗^, *Atp5sl* ^∗^, *Abca9*, *Samm50* ^∗^, *Mtx1* ^∗^, *Hk2* ^∗^, *Sfxn4*, *Hk1* ^∗^, *Letm2* ^∗^, *Wars2* ^∗^, *Bcl2l1* ^∗^, *Mrpl11* ^∗^, *Ivd* ^∗^, *Slc25a46* ^∗^, *Etfb* ^∗^, *Pdk2* ^∗^, *Mobp*, *Tomm40* ^∗^, *Atad1* ^∗^, *Acsm3*, *Mrpl22* ^∗^, *Adap2*, *Hmgcs2* ^∗^, *Tfrc*, *Ndufv1* ^∗^, *Gls* ^∗^, *Bola1* ^∗^, *Alkbh3* ^∗^, *Acad11* ^∗^, *Dap3* ^∗^, *Comtd1* ^∗^
	Mitochondrial outer membrane	11	100	*Cyb5r3* ^∗^, *Mfn1* ^∗^, *Samm50* ^∗^, *Mtx1* ^∗^, *Hk2* ^∗^, *Hk1* ^∗^, *Tomm40* ^∗^, *Bnip3* ^∗^, *Bcl2l1* ^∗^, *Cyb5b* ^∗^, *Synj2bp* ^∗^
	Mitochondrial inner membrane	15	80	*Cyb5r3* ^∗^, *Atp5d* ^∗^, *Uqcrc1* ^∗^, *Samm50* ^∗^, *Letm2* ^∗^, *Cyb5b* ^∗^, *Hmgcs2* ^∗^, *Slc25a34*, *Ndufv1* ^∗^, *Dnajc11* ^∗^, *Slc25a22* ^∗^, *Atp5c1* ^∗^, *Slc25a46* ^∗^, *Abcb10*, *Atp5h*
	Mitochondrial matrix	12	92	*Mrpl11* ^∗^, *Atp5d* ^∗^, *Acsm3*, *Pdk2* ^∗^, *Fech* ^∗^, *Hmgcs2* ^∗^, *Ivd* ^∗^, *Gls* ^∗^, *Atp5c1* ^∗^, *Wars2* ^∗^, *Etfb* ^∗^, *Dap3* ^∗^

^∗^DEGs that SkQ1 eliminated in OXYS rats. BP: biological process, MF: molecular function, CC: cellular component.

## Data Availability

The data used to support the findings of this study are available from the corresponding author upon request.

## Supplementary Materials

Supplementary Materials Supplementary Data 1: DEGs (122 genes) in untreated OXYS rats and 13 genes in SkQ1-treated OXYS rats were associated with mitochondrial function. Treatment with SkQ1 changed the expression of genes specific to neurons, astrocytes, oligodendrocytes, microglia, and endothelial cells in OXYS rats.

## References

[1] Cheignon C., Tomas M., Bonnefont-Rousselot D., Faller P., Hureau C., Collin F. (2018). Oxidative stress and the amyloid beta peptide in Alzheimer’s disease. *Redox Biology*.

[2] Persson T., Popescu B. O., Cedazo-Minguez A. (2014). Oxidative stress in Alzheimer’s disease: why did antioxidant therapy fail?. *Oxidative Medicine and Cellular Longevity*.

[3] Liu Z., Zhou T., Ziegler A. C., Dimitrion P., Zuo L. (2017). Oxidative stress in neurodegenerative diseases: from molecular mechanisms to clinical applications. *Oxidative Medicine and Cellular Longevity*.

[4] Kryscio R. J., Abner E. L., Caban-Holt A. (2017). Association of antioxidant supplement use and dementia in the Prevention of Alzheimer’s Disease by Vitamin E and Selenium Trial (PREADViSE). *JAMA Neurology*.

[5] Mattson M. P. (2004). Pathways towards and away from Alzheimer’s disease. *Nature*.

[6] Frere S., Slutsky I. (2018). Alzheimer’s disease: from firing instability to homeostasis network collapse. *Neuron*.

[7] Feng Y., Wang X. (2012). Antioxidant therapies for Alzheimer’s disease. *Oxidative Medicine and Cellular Longevity*.

[8] Skulachev V. P. (2012). Mitochondria-targeted antioxidants as promising drugs for treatment of age-related brain diseases. *Journal of Alzheimer's Disease*.

[9] Skulachev V. P., Anisimov V. N., Antonenko Y. N. (2009). An attempt to prevent senescence: a mitochondrial approach. *Biochimica et Biophysica Acta (BBA) - Bioenergetics*.

[10] Stefanova N. A., Fursova A. Z., Kolosova N. G. (2010). Behavioral effects induced by mitochondria-targeted antioxidant SkQ1 in Wistar and senescence-accelerated OXYS rats. *Journal of Alzheimer's Disease*.

[11] Stefanova N. A., Muraleva N. A., Skulachev V. P., Kolosova N. G. (2014). Alzheimer’s disease-like pathology in senescence-accelerated OXYS rats can be partially retarded with mitochondria-targeted antioxidant SkQ1. *Journal of Alzheimer's Disease*.

[12] Stefanova N. A., Muraleva N. A., Maksimova K. Y. (2016). An antioxidant specifically targeting mitochondria delays progression of Alzheimer’s disease-like pathology. *Aging*.

[13] Loshchenova P. S., Sinitsyna O. I., Fedoseeva L. A., Stefanova N. A., Kolosova N. G. (2015). Influence of antioxidant SkQ1 on accumulation of mitochondrial DNA deletions in the hippocampus of senescence-accelerated OXYS rats. *Biochemistry (Moscow)*.

[14] Kolosova N. G., Tyumentsev M. A., Muraleva N. A., Kiseleva E., Vitovtov A. O., Stefanova N. A. (2017). Antioxidant SkQ1 alleviates signs of Alzheimer’s disease-like pathology in old OXYS rats by reversing mitochondrial deterioration. *Current Alzheimer Research*.

[15] Stefanova N., Kozhevnikova O., Vitovtov A. (2014). Senescence-accelerated OXYS rats: a model of age-related cognitive decline with relevance to abnormalities in Alzheimer disease. *Cell Cycle*.

[16] Stefanova N. A., Muraleva N. A., Korbolina E. E., Kiseleva E., Maksimova K. Y., Kolosova N. G. (2015). Amyloid accumulation is a late event in sporadic Alzheimer’s disease-like pathology in nontransgenic rats. *Oncotarget*.

[17] Stefanova N. A., Maksimova K. Y., Rudnitskaya E. A., Muraleva N. A., Kolosova N. G. (2018). Association of cerebrovascular dysfunction with the development of Alzheimer’s disease-like pathology in OXYS rats. *BMC Genomics*.

[18] Stefanova N. A., Ershov N. I., Maksimova K. Y., Muraleva N. A., Tyumentsev M. A., Kolosova N. G. (2019). The rat prefrontal-cortex transcriptome: effects of aging and sporadic Alzheimer’s disease–like pathology. *The Journals of Gerontology: Series A*.

[19] Rudnitskaya E. A., Kolosova N. G., Stefanova N. A. (2017). Impact of changes in neurotrophic supplementation on development of Alzheimer’s disease-like pathology in OXYS rats. *Biochemistry*.

[20] Tyumentsev M. A., Stefanova N. A., Muraleva N. A. (2018). Mitochondrial dysfunction as a predictor and driver of Alzheimer’s disease-like pathology in OXYS rats. *Journal of Alzheimer’s Disease*.

[21] Sutherland G. T., Janitz M., Kril J. J. (2011). Understanding the pathogenesis of Alzheimer’s disease: will RNA-Seq realize the promise of transcriptomics?. *Journal of Neurochemistry*.

[22] Kim D., Pertea G., Trapnell C., Pimentel H., Kelley R., Salzberg S. L. (2013). TopHat2: accurate alignment of transcriptomes in the presence of insertions, deletions and gene fusions. *Genome Biology*.

[23] Love M. I., Huber W., Anders S. (2014). Moderated estimation of fold change and dispersion for RNA-seq data with DESeq2. *Genome Biology*.

[24] Calvo S. E., Clauser K. R., Mootha V. K. (2016). MitoCarta2.0: an updated inventory of mammalian mitochondrial proteins. *Nucleic Acids Research*.

[25] Zhang Y., Chen K., Sloan S. A. (2014). An RNA-sequencing transcriptome and splicing database of glia, neurons, and vascular cells of the cerebral cortex. *Journal of Neuroscience*.

[26] Anders S., Huber W. (2010). Differential expression analysis for sequence count data. *Genome Biology*.

[27] Stefanova N. A., Maksimova K. Y., Kiseleva E., Rudnitskaya E. A., Muraleva N. A., Kolosova N. G. (2015). Melatonin attenuates impairments of structural hippocampal neuroplasticity in OXYS rats during active progression of Alzheimer’s disease-like pathology. *Journal of Pineal Research*.

[28] Heneka M. T., Golenbock D. T., Latz E. (2015). Innate immunity in Alzheimer’s disease. *Nature Immunology*.

[29] Heppner F. L., Ransohoff R. M., Becher B. (2015). Immune attack: the role of inflammation in Alzheimer disease. *Nature Reviews Neuroscience*.

[30] Zabel M., Nackenoff A., Kirsch W. M., Harrison F. E., Perry G., Schrag M. (2018). Markers of oxidative damage to lipids, nucleic acids and proteins and antioxidant enzymes activities in Alzheimer’s disease brain: a meta-analysis in human pathological specimens. *Free Radical Biology and Medicine*.

[31] Gornicka A., Morris-Stiff G., Thapaliya S., Papouchado B. G., Berk M., Feldstein A. E. (2011). Transcriptional profile of genes involved in oxidative stress and antioxidant defense in a dietary murine model of steatohepatitis. *Antioxidants and Redox Signaling*.

[32] Ahn J. H., Chen B. H., Shin B.-N. (2016). Comparison of catalase immunoreactivity in the hippocampus between young, adult and aged mice and rats. *Molecular Medicine Reports*.

[33] Miyashita A., Hatsuta H., Kikuchi M. (2014). Genes associated with the progression of neurofibrillary tangles in Alzheimer’s disease. *Translational Psychiatry*.

[34] Guan P. P., Wang P. (2018). Integrated communications between cyclooxygenase-2 and Alzheimer’s disease. *The FASEB Journal*.

[35] Kolosova N. G., Shcheglova T. V., Sergeeva S. V., Loskutova L. V. (2006). Long-term antioxidant supplementation attenuates oxidative stress markers and cognitive deficits in senescent-accelerated OXYS rats. *Neurobiology of Aging*.

[36] Kolosova N. G., Stefanova N. A., Korbolina E. E., Fursova A. Z., Kozhevnikova O. S. (2014). Senescence-accelerated OXYS rats: a genetic model of premature aging and age-related diseases. *Advances in Gerontology*.

[37] Maksimova K. Y., Logvinov S. V., Stefanova N. A. (2014). Ultrastructure of synapses in the hippocampus of rats in aging. *Bulletin of Siberian Medicine*.

[38] Kuo T. Y., Chen C. Y., Hsueh Y. P. (2010). Bcl11A/CTIP1 mediates the effect of the glutamate receptor on axon branching and dendrite outgrowth. *Journal of Neurochemistry*.

[39] Popugaeva E., Pchitskaya E., Bezprozvanny I. (2017). Dysregulation of neuronal calcium homeostasis in Alzheimer’s disease - a therapeutic opportunity?. *Biochemical and Biophysical Research Communications*.

[40] Palmer C. L., Lim W., Hastie P. G. R. (2005). Hippocalcin functions as a calcium sensor in hippocampal LTD. *Neuron*.

[41] Kettenmann H., Kirchhoff F., Verkhratsky A. (2013). Microglia: new roles for the synaptic stripper. *Neuron*.

[42] Chung W. S., Welsh C. A., Barres B. A., Stevens B. (2015). Do glia drive synaptic and cognitive impairment in disease?. *Nature Neuroscience*.

[43] Yan Y., Tan X., Wu X. (2013). Involvement of early growth response-2 (Egr-2) in lipopolysaccharide-induced neuroinflammation. *Journal of Molecular Histology*.

[44] Gómez Ravetti M., Rosso O. A., Berretta R., Moscato P. (2010). Uncovering molecular biomarkers that correlate cognitive decline with the changes of hippocampus’ gene expression profiles in Alzheimer’s disease. *PLoS One*.

[45] Yin Z., Raj D., Saiepour N. (2017). Immune hyperreactivity of A*β* plaque-associated microglia in Alzheimer’s disease. *Neurobiology of Aging*.

[46] Desjardins S., Mayo W., Vallée M. (1997). Effect of aging on the basal expression of c-Fos, c-Jun, and Egr-1 proteins in the hippocampus. *Neurobiology of Aging*.

[47] Killick R., Ribe E. M., al-Shawi R. (2014). Clusterin regulates *β*-amyloid toxicity via Dickkopf-1-driven induction of the wnt–PCP–JNK pathway. *Molecular Psychiatry*.

[48] Schmidt C., Frahm C., Schneble N. (2016). Phosphoinositide 3-kinase *γ* restrains neurotoxic effects of microglia after focal brain ischemia. *Molecular Neurobiology*.

[49] Kim J. I., Lee H. R., Sim S. E. (2011). PI3K*γ* is required for NMDA receptor-dependent long-term depression and behavioral flexibility. *Nature Neuroscience*.

[50] Zlokovic B. V. (2011). Neurovascular pathways to neurodegeneration in Alzheimer’s disease and other disorders. *Nature Reviews Neuroscience*.

[51] Tarantini S., Tran C. H. T., Gordon G. R., Ungvari Z., Csiszar A. (2017). Impaired neurovascular coupling in aging and Alzheimer’s disease: contribution of astrocyte dysfunction and endothelial impairment to cognitive decline. *Experimental Gerontology*.

[52] Agafonova I. G., Kolosova N. G., Mishchenko N. P., Chaikina E. L., Stonik V. A. (2007). Effect of histochrome on brain vessels and research and exploratory activity of senescence-accelerated OXYS rats. *Bulletin of Experimental Biology and Medicine*.

[53] Agafonova I. G., Kotelnikov V. N., Kolosova N. G., Stonik V. A. (2012). Comparative study on hypertension-induced cerebral vascular alterations in two rat lines by magnetic resonance angiography. *Applied Magnetic Resonance*.

[54] Maslov M. Y., Chernysheva G. A., Smol’jakova V. I., Aliev O. I., Kolosova N. G., Plotnikov M. B. (2015). Hemorheological parameters and their correlations in OXYS rats: a new model of hyperviscosity syndrome. *Clinical Hemorheology and Microcirculation*.

[55] Chang C. Y., Liang H. J., Chow S. Y., Chen S. M., Liu D. Z. (2007). Hemorheological mechanisms in Alzheimer’s disease. *Microcirculation*.

[56] De-Paula V. J., Radanovic M., Diniz B. S., Forlenza O. V. (2012). Alzheimer’s disease. *Subcellular Biochemistry*.

